# Supplementary material for: Essential and Toxic Mineral Content and Fatty Acid Profile of Colostrum in Dairy Sheep
Source: Animals (Basel). 2022 Oct 11;12(20):2730. doi: 10.3390/ani12202730 (PMC9597829; doi:10.3390/ani12202730)
Supplement: Supplementary file 1 [file animals-12-02730-s001.zip › animals-1852736-supplementary.pdf]

animals

MDPI

Essential and toxic minerals content and fatty acid profile of colostrum in dairy sheep

Maria Francesca Guiso [1], Gianni Battacone [1], Linda Canu [1], Mario Deroma[1], Ilaria Langasco [2], Gavino Sanna [2], Eleni Tsiplakou[3], Giuseppe Pulina [1], and Anna Nudda [1,*]

Supplementary Material

Table S1. Microwave-assisted procedure of mineralization of sheep's colostrum and milk samples.

**Content of each vessel**	Sample weight: ca. 1 g (exactly weighted, uncertainty: 0.0001 g); HNO_3 67%: 2 cm^3; H_2O_2 30%: 2 cm^3; ultrapure water: 7 cm^3
**Heating step**	From room temperature to 200°C in 15 minutes, power: 1800W
**Standing step**	200°C for 15 minutes, power: 1800W
**Cooling step**	From 200°C to room temperature in 15 minutes.

Table S2. ICP-MS instrumental settings for the determination of oligoelements and toxic element in sheep's colostrum and milk.

**RF power generator (W)**	1300	**KED gas**	Helium, 99.999%
**Ar plasma flow (dm^3 min^{-1})**	17.995	**Masses of optimization**	^{7}Li, ^{89}Y and ^{205}Tl
**Ar auxiliary flow (dm^3 min^{-1})**	1.203	**Dwell time (ms)**	50
**Ar nebulizer flow (dm^3 min^{-1})**	0.991	**Number of points per peak**	3
**Nebulizer**	Meinhard®, glass	**Acquisition time (s)**	3
**Spray chamber**	Cyclonic, glass	**Quantification**	External calibration
**Skimmer and sampling cones**	Nickel	**KED gas flow (cm^3 min^{-1})**	3.5
**Sampling depth (mm)**	0	**Masses of optimization**	^{7}Li, ^{89}Y and ^{205}Tl

**Element**	**Quantification isotopic ion (% elemental abundance)**	**Interferents**	**Analysing mode**	**Correction equation**
**Cd**	$^{111}Cd^{+}$ (12.80)	$^{95}Mo^{16}O^{+}$	Normal	
**Cu**	$^{63}Cu^{+}$ (69.17)	$^{31}P^{16}O_2^{+}$; $^{47}Ti^{16}O^{+}$; $^{126}Te^{2+}$	Normal	
**Mn**	$^{55}Mn^{+}$ (100)	$^{40}Ar^{14}N^{1}H^{+}$;$^{1}H^{37}Cl^{17}O^{+}$;$^{37}Cl^{18}O^{+}$	Normal	
**Ni**	$^{60}Ni^{+}$ (26.22)	$^{44}Ca^{16}O^{+}$	Normal	
**Pb**	$^{208}Pb^{+}$ (52.40)		Normal	

**Se**	$^{82}Se^{+}$ (8.73)	$^{82}Kr^{+}$; $^{81}Br^{1}H^{+}$; $^{40}Ar_2H^{+}$; Ho^{2+}; Gd^{2+}; Er^{2+}	KED	-0.00783x^{83}Kr
**Zn**	$^{66}Zn^{+}$ (27.90)	$^{50}Ti^{16}O^{+}$, $^{50}V^{16}O^{+}$, $^{34}S^{16}O_2^{+}$, $^{132}Ba^{2+}$	Normal	

Table S3. Validation parameters for the determination of oligoelements and toxic element in sheep's colostrum and milk.

	**Sensitivity**			**Linearity**		**Precision**		**Trueness**	**CRM Certified concentration**
Element	LoD (μg dm^{-3})	LoQ (μg dm^{-3})	Range (μg kg^{-1})	Regression line	Correlation coefficient R^2	Repeatability[a] (CV)	Intermediate precision[b] (CV)	Recovery (%)	(mg kg^{-1})
**Cd**	0.0002	0.0007	0.04-40	y = (4920±10) x + (491±20)	1	4.1[c]	18[c]	110[c]	*0.002*[c]
						4.2[d]	8.3[d]	98[d]	0.106[d]
**Cu**	0.014	0.048	0.2-200	y = (20800±100) x + (8190±40)	1	9.7[c]	7.7[c]	105[c]	4.3[c]
						5.0[d]	2.7[d]	90[d]	5.0[d]
**Mn**	0.005	0.018	0.15-150	y = (68570±200) x + (14270±150)	1	3.0[c]	3.6[c]	107[c]	*1.04*[c]
						8.0[d]	6.9[d]	102[d]	0.29[d]
**Ni**	0.0008	0.0027	0.25-250	y = (10490±300) x – (4610±100)	1	7.1[c]	8.4[c]	10[c]	*0.011*[c]
						8.0[d]	9.0[d]	107[d]	*0.28*[d]
**Pb**	0.001	0.003	0.09-90	y = (27720±250) x – (5510±200)	0.9999	3.9[c]	14[c]	113[c]	*0.12*[c]
						2.7[d]	3.6[d]	91[d]	0.207[d]
**Se**	0.0008	0.0026	0.1-100	y = (25±5) x + (20±1)	0.9999	3.7[c]	4.2[c]	100[c]	0.24[c]
						4.6[d]	11[d]	95[d]	0.19[d]
**Zn**	0.006	0.021	0.35-350	y = (5310±50)x + (19260±400)	1	4.4[c]	2.5[c]	97[c]	13[c]
						2.3[d]	5.6[d]	95[d]	44.9[d]

LoD, limit of detection; LoQ, limit of quantification; CV, coefficient of variation; [a] parameter evaluated repeating the analysis of a CRM for five times within the same analytical session; [b] parameter evaluated repeating the analysis of a CRM on five different analytical sessions performed within one month; [c] measured on IAEA-A-13 certified reference sample, [d] measured on ERM-BD151 certified reference sample; CRM concentration in *italics* is a not certified data; n = 5.

Table S4. Average concentration (% on total amount of FAs) of all FAs measured in colostrum of each of the eight farms

	Farms									
	**A**	**B**	**C**	**D**	**E**	**F**	**G**	**H**	**P value**	**SEM**
**Fat, %**	5.3 ^{c}	7.6 ^{abc}	7.7 ^{abc}	9.6 ^{ab}	6.9 ^{abc}	5.5 ^{bc}	9.1 ^{abc}	10.2 ^{a}	0.0013	0.3575
**FA (% on Total FAs)**										
Short chain FA										
**C4:0**	1.93 ^{ab}	1.96 ^{ab}	1.70 ^{b}	1.83 ^{bc}	2.22 ^{a}	1.99 ^{ab}	1.66 ^{b}	1.60 ^{b}	0.0041	0.0427
**C6:0**	0.69 ^{b}	0.84 ^{b}	0.73 ^{b}	0.67 ^{b}	1.13 ^{a}	0.60 ^{b}	0.71 ^{b}	0.58 ^{b}	<0.0001	0.0270
**C7:0**	0.04 ^{bc}	0.04 ^{bc}	0.03 ^{c}	0.05 ^{bc}	0.05 ^{bc}	0.12 ^{a}	0.05 ^{b}	0.04 ^{c}	<0.0001	0.0027
**C8:0**	0.49 ^{b}	0.66 ^{b}	0.57 ^{b}	0.50 ^{b}	0.92 ^{a}	0.39 ^{b}	0.53 ^{b}	0.43 ^{b}	<0.0001	0.0259
**C9:0**	0.08 ^{b}	0.10 ^{b}	0.08 ^{b}	0.10 ^{b}	0.11 ^{b}	0.22 ^{a}	0.10 ^{b}	0.08 ^{b}	<0.0001	0.0053
**C10:0**	1.54 ^{bc}	2.14 ^{b}	1.78 ^{bc}	1.55 ^{bc}	3.04 ^{a}	1.23 ^{bc}	1.68 ^{bc}	1.28 ^{c}	<0.0001	0.0885
**C10:1**	0.07 ^{ab}	0.06 ^{abc}	0.06 ^{abc}	0.07 ^{ab}	0.08 ^{ab}	0.03 ^{c}	0.05 ^{bc}	0.08 ^{a}	<0.0001	0.0028
Medium chain FA										
**C11:0**	0.11 ^{bc}	0.14 ^{b}	0.11 ^{bc}	0.12 ^{bc}	0.22 ^{a}	0.09 ^{bc}	0.10 ^{bc}	0.09 ^{c}	<0.0001	0.0063
**C12:0**	1.98 ^{b}	2.12 ^{b}	2.01 ^{b}	2.13 ^{b}	2.74 ^{a}	1.72 ^{b}	1.93 ^{b}	1.77 ^{b}	<0.0001	0.0509
**isoC13:0**	0.01	0.01	0.01	0.01	0.01	0.01	0.01	0.01	0.4431	0.0004
**anteisoC13:0**	0.03 ^{b}	0.03 ^{ab}	0.03 ^{b}	0.04 ^{ab}	0.05 ^{a}	0.03 ^{b}	0.03 ^{b}	0.03 ^{b}	0.0003	0.0013
**isoC14:0**	0.06 ^{ab}	0.06 ^{ab}	0.06 ^{ab}	0.05 ^{ab}	0.05 ^{ab}	0.05 ^{ab}	0.07 ^{a}	0.04 ^{b}	0.0352	0.0018
**C14:0**	11.01 ^{bc}	11.75 ^{ab}	9.96 ^{bc}	11.78 ^{ab}	14.35 ^{a}	10.62 ^{bc}	8.86 ^{c}	9.19 ^{bc}	<0.0001	0.2857
**isoC15:0**	0.19	0.19	0.16	0.17	0.16	0.20	0.19	0.17	0.0188	0.0038
**anteisoC15:0**	0.20	0.23	0.17	0.20	0.22	0.21	0.22	0.17	0.0561	0.0055
**C14:1c9**	0.39 ^{b}	0.47 ^{ab}	0.26 ^{b}	0.48 ^{ab}	0.73 ^{a}	0.42 ^{b}	0.23 ^{b}	0.31 ^{b}	<0.0001	0.0258
**C15:0**	0.55 ^{bc}	0.64 ^{ab}	0.52 ^{bc}	0.52 ^{bc}	0.72 ^{a}	0.49 ^{bc}	0.61 ^{abc}	0.47 ^{c}	<0.0001	0.0150
**C15:1**	0.03 ^{b}	0.03 ^{b}	0.03 ^{b}	0.02 ^{b}	0.05 ^{a}	0.03 ^{b}	0.03 ^{b}	0.03 ^{b}	<0.0001	0.0013
**isoC16:0**	0.20 ^{ab}	0.21 ^{ab}	0.19 ^{ab}	0.19 ^{ab}	0.19 ^{ab}	0.21 ^{ab}	0.22 ^{a}	0.17 ^{b}	0.0473	0.0040
**C16:0**	27.19 ^{bc}	31.47 ^{b}	25.67 ^{c}	29.11 ^{bc}	37.12 ^{a}	27.92 ^{bc}	25.56 ^{c}	25.84 ^{c}	<0.0001	0.5818
**isoC17:0**	0.42 ^{bc}	0.40 ^{cd}	0.41 ^{bcd}	0.39 ^{cd}	0.34 ^{d}	0.49 ^{ab}	0.50 ^{a}	0.43 ^{abc}	<0.0001	0.0078
**anteisoC17:0**	0.46 ^{b}	0.45 ^{bc}	0.44 ^{bc}	0.45 ^{bc}	0.36 ^{c}	0.51 ^{ab}	0.57 ^{a}	0.51 ^{ab}	<0.0001	0.0097
**C16:1c9**	1.38 ^{b}	1.85 ^{ab}	1.08 ^{b}	1.78 ^{ab}	2.63 ^{a}	1.72 ^{ab}	1.08 ^{b}	1.24 ^{b}	<0.0001	0.0872
**C17:0**	0.81 ^{b}	0.86 ^{ab}	0.83 ^{b}	0.78 ^{b}	0.68 ^{b}	0.84 ^{ab}	1.03 ^{a}	0.87 ^{ab}	0.0003	0.0185
**isoC18:0**	0.11 ^{bc}	0.12 ^{ab}	0.12 ^{bc}	0.12 ^{bc}	0.08 ^{c}	0.12 ^{abc}	0.15 ^{a}	0.13 ^{ab}	<0.0001	0.0035
**C17:1c9**	0.43 ^{bc}	0.43 ^{bc}	0.46 ^{ab}	0.45 ^{abc}	0.33 ^{c}	0.48 ^{ab}	0.54 ^{a}	0.48 ^{ab}	<0.0001	0.0107
Long chain FA										
**C18:0**	7.51 ^{ab}	7.29 ^{ab}	8.06 ^{a}	7.19 ^{ab}	5.02 ^{b}	6.64 ^{ab}	7.78 ^{a}	8.18 ^{a}	0.0130	0.2206
**C18:1t9**	0.22 ^{ab}	0.17 ^{c}	0.26 ^{a}	0.20 ^{bc}	0.15 ^{c}	0.21 ^{abc}	0.23 ^{ab}	0.24 ^{ab}	<0.0001	0.0057
**C18:1t10**	0.24	0.23	0.34	0.20	0.30	0.20	0.29	0.25	0.4630	0.0183
**C18:1t11**	0.81 ^{a}	0.59 ^{ab}	0.78 ^{ab}	0.56 ^{ab}	0.49 ^{b}	0.82 ^{ab}	0.71 ^{ab}	0.57 ^{ab}	0.0068	0.0276
**C18:1t12**	0.27 ^{ab}	0.16 ^{c}	0.31 ^{a}	0.20 ^{bc}	0.18 ^{c}	0.24 ^{abc}	0.27 ^{ab}	0.23 ^{abc}	<0.0001	0.0083
**C18:1t13:t14**	0.36 ^{a}	0.25 ^{ab}	0.30 ^{ab}	0.29 ^{ab}	0.34 ^{ab}	0.33 ^{ab}	0.28 ^{ab}	0.22 ^{b}	0.0350	0.0118
**C18:1c9**	30.52 ^{ab}	25.99 ^{b}	32.68 ^{a}	29.40 ^{ab}	17.18 ^{c}	31.26 ^{ab}	33.35 ^{a}	35.76 ^{a}	<0.0001	0.7795
**C18:2n6 (LA)**	2.24 ^{b}	2.21 ^{b}	2.92 ^{ab}	2.43 ^{b}	2.34 ^{b}	2.08 ^{b}	3.24 ^{b}	2.70 ^{ab}	0.0002	0.0744
**C20:0**	0.23 ^{ab}	0.22 ^{ab}	0.22 ^{ab}	0.19 ^{bc}	0.17 ^{c}	0.26 ^{a}	0.26 ^{a}	0.19 ^{bc}	<0.0001	0.0051
**C18:3n6**	0.04 ^{b}	0.05 ^{a}	0.05 ^{ab}	0.04 ^{ab}	0.05 ^{ab}	0.04 ^{b}	0.05 ^{ab}	0.05 ^{ab}	0.0107	0.0014
**C20:1c9**	0.04 ^{a}	0.03 ^{abc}	0.03 ^{abc}	0.03 ^{bc}	0.02 ^{c}	0.03 ^{abc}	0.03 ^{ab}	0.02 ^{c}	0.0001	0.0009
**C18:3n3 (ALA)**	0.61 ^{a}	0.46 ^{bcd}	0.33 ^{de}	0.37 ^{cd}	0.41 ^{bcd}	0.56 ^{ab}	0.49 ^{abc}	0.21 ^{e}	<0.0001	0.0170
**CLAc9t11**	0.89 ^{a}	0.56 ^{cd}	0.81 ^{ab}	0.61 ^{bcd}	0.47 ^{d}	0.92 ^{a}	0.71 ^{abc}	0.58 ^{cd}	<0.0001	0.0239
**CLAt10c12**	0.03 ^{ab}	0.03 ^{bc}	0.03 ^{bc}	0.02 ^{cd}	0.02 ^{cd}	0.04 ^{a}	0.03 ^{cd}	0.02 ^{d}	<0.0001	0.0009
**CLAt12t14**	0.02 ^{a}	0.01 ^{c}	0.01 ^{abc}	0.01 ^{bc}	0.01 ^{bc}	0.02 ^{a}	0.01 ^{ab}	0.01 ^{c}	<0.0001	0.0005
**CLAt11t13**	0.03 ^{ab}	0.04 ^{a}	0.03 ^{ab}	0.03 ^{ab}	0.03 ^{ab}	0.04 ^{ab}	0.03 ^{b}	0.03 ^{b}	0.011	0.0009
**CLAt9t11**	0.02 ^{a}	0.02 ^{ab}	0.02 ^{b}	0.02 ^{b}	0.02 ^{ab}	0.02 ^{ab}	0.02 ^{b}	0.01 ^{b}	<0.0001	0.0007
**C18:4n3**	0.01	0.01	0.01	0.01	0.01	0.01	0.01	0.01	0.3227	0.0003

**C20:2n6**	0.02	0.03	0.03	0.03	0.03	0.02	0.03	0.03	0.2705	0.0007
**C20:3n9**	0.07	0.07	0.06	0.08	0.06	0.08	0.07	0.06	0.0284	0.0016
**C22:0**	0.07 ^{ab}	0.07 ^{ab}	0.06 ^{ab}	0.05 ^{b}	0.06 ^{ab}	0.07 ^{ab}	0.09 ^{a}	0.05 ^{b}	0.0013	0.0027
**C20:3n6**	0.03 ^{b}	0.03 ^{ab}	0.04 ^{a}	0.04 ^{ab}	0.04 ^{ab}	0.03 ^{b}	0.03 ^{ab}	0.04 ^{ab}	0.0009	0.0008
**C20:4n6 (ARA)**	0.26 ^{c}	0.27 ^{bc}	0.33 ^{ab}	0.32 ^{abc}	0.28 ^{bc}	0.24 ^{c}	0.39 ^{a}	0.37 ^{a}	<0.0001	0.0081
**C23:0**	0.02	0.03	0.02	0.02	0.02	0.02	0.03	0.01	0.0579	0.0013
**EPA**	0.10 ^{a}	0.08 ^{b}	0.07 ^{b}	0.07 ^{bc}	0.07 ^{b}	0.08 ^{ab}	0.07 ^{b}	0.05 ^{c}	<0.0001	0.0023
**DPA**	0.24 ^{a}	0.16 ^{bc}	0.18 ^{bc}	0.14 ^{cd}	0.16 ^{bcd}	0.20 ^{ab}	0.20 ^{ab}	0.11 ^{d}	<0.0001	0.0059
**DHA**	0.09 ^{a}	0.06 ^{b}	0.06 ^{b}	0.05 ^{bc}	0.06 ^{b}	0.06 ^{ab}	0.06 ^{b}	0.03 ^{c}	<0.0001	0.0024
Groups of FA										
**SCFA**	4.84 ^{bc}	5.80 ^{b}	4.96 ^{bc}	4.77 ^{bc}	7.55 ^{a}	4.59 ^{bc}	4.79 ^{bc}	4.09 ^{c}	<0.0001	0.1653
**MCFA**	46.47 ^{bc}	52.23 ^{b}	43.35 ^{c}	49.58 ^{bc}	61.79 ^{a}	47.02 ^{bc}	42.77 ^{c}	42.73 ^{c}	<0.0001	0.9431
**LCFA**	48.69 ^{ab}	41.97 ^{b}	51.68 ^{a}	45.65 ^{ab}	30.66 ^{c}	48.40 ^{ab}	52.44 ^{a}	53.18 ^{a}	<0.0001	1.0364
**SFA**	56.17 ^{bc}	62.25 ^{b}	54.15 ^{c}	58.41 ^{bc}	70.21 ^{a}	55.27 ^{bc}	53.16 ^{c}	52.50 ^{c}	<0.0001	0.8262
**MUFA**	37.62 ^{ab}	32.72 ^{b}	39.57 ^{a}	36.27 ^{ab}	24.82 ^{c}	38.73 ^{ab}	40.07 ^{a}	42.17 ^{a}	<0.0001	0.7501
**PUFA**	6.19 ^{ab}	5.01 ^{b}	6.27 ^{ab}	5.31 ^{b}	4.95 ^{b}	5.99 ^{ab}	6.75 ^{a}	5.32 ^{b}	<0.0001	0.1261
**UFA**	43.81 ^{ab}	37.74 ^{b}	45.83 ^{a}	41.58 ^{ab}	29.78 ^{c}	44.72 ^{ab}	46.82 ^{a}	47.49 ^{a}	<0.0001	0.8264
**OCFA**	1.62 ^{bc}	1.82 ^{ab}	1.60 ^{bc}	1.60 ^{bc}	1.82 ^{abc}	1.80 ^{abc}	1.94 ^{a}	1.56 ^{c}	<0.0001	0.0240
**BCFA**	1.69 ^{bc}	1.71 ^{bc}	1.59 ^{bc}	1.62 ^{bc}	1.47 ^{c}	1.83 ^{ab}	1.97 ^{a}	1.66 ^{bc}	<0.0001	0.0253
**OBCFA**	3.32 ^{b}	3.53 ^{ab}	3.19 ^{b}	3.22 ^{b}	3.28 ^{b}	3.63 ^{ab}	3.91 ^{a}	3.22 ^{b}	<0.0001	0.0434
**PUFA6**	2.67 ^{bc}	2.67 ^{bc}	3.45 ^{ab}	2.95 ^{bc}	2.81 ^{bc}	2.47 ^{c}	3.83 ^{a}	3.28 ^{abc}	<0.0001	0.0818
**PUFA3**	1.07 ^{a}	0.80 ^{bcd}	0.67 ^{cd}	0.64 ^{d}	0.74 ^{bcd}	0.94 ^{ab}	0.84 ^{bc}	0.42 ^{e}	<0.0001	0.0257
**n6/n3**	2.50 ^{e}	3.31 ^{cd}	5.20 ^{b}	4.61 ^{b}	3.77 ^{c}	2.63 ^{de}	4.68 ^{b}	7.76 ^{a}	<0.0001	0.1847
**n3/n6**	0.40 ^{a}	0.31 ^{b}	0.19 ^{d}	0.22 ^{cd}	0.27 ^{bc}	0.38 ^{a}	0.22 ^{d}	0.13 ^{e}	<0.0001	0.0104
**CLA**	1.05 ^{a}	0.70 ^{cd}	0.94 ^{ab}	0.73 ^{bcd}	0.60 ^{d}	1.09 ^{a}	0.84 ^{abc}	0.68 ^{cd}	<0.0001	0.0255
**TFA**	4.01 ^{a}	2.74 ^{d}	3.94 ^{ab}	2.97 ^{bcd}	2.77 ^{cd}	3.90 ^{abc}	3.63 ^{abcd}	3.07 ^{abcd}	<0.0001	0.0980
**TFA (without VA)**	3.20 ^{a}	2.15 ^{c}	3.16 ^{a}	2.41 ^{bc}	2.28 ^{bc}	3.08 ^{ab}	2.92 ^{ab}	2.50 ^{abc}	<0.0001	0.0724
**AI**	1.70 ^{bc}	2.22 ^{b}	1.52 ^{bc}	2.01 ^{bc}	3.45 ^{a}	1.69 ^{bc}	1.35 ^{c}	1.39 ^{c}	<0.0001	0.0945
**TI**	1.63 ^{bc}	2.20 ^{b}	1.55 ^{bc}	2.00 ^{bc}	3.33 ^{a}	1.68 ^{bc}	1.41 ^{c}	1.48 ^{bc}	<0.0001	0.0883
**h/H**	0.94 ^{ab}	0.72 ^{bc}	1.08 ^{c}	0.87 ^{ab}	0.44 ^{c}	0.97 ^{ab}	1.13 ^{a}	1.17 ^{a}	<0.0001	0.0357
**DI C10:1**	4.23 ^{ab}	3.12 ^{b}	3.67 ^{b}	4.26 ^{ab}	2.54 ^{b}	2.69 ^{b}	2.80 ^{b}	5.96 ^{a}	<0.0001	0.1812
**DI C14:1**	3.25 ^{ab}	3.75 ^{ab}	2.52 ^{b}	3.78 ^{ab}	4.62 ^{a}	3.48 ^{ab}	2.51 ^{b}	3.10 ^{b}	0.0001	0.1279
**DI C16:1**	4.79 ^{ab}	5.41 ^{ab}	4.01 ^{b}	5.46 ^{ab}	6.42 ^{a}	5.48 ^{ab}	4.03 ^{b}	4.54 ^{b}	0.0004	0.1554
**DI C18:1**	80.43	77.93	79.94	80.00	77.95	82.69	81.06	81.28	0.2057	0.4639
**DI CLA**	52.81	49.25	50.85	52.43	51.59	54.18	49.96	51.32	0.7001	0.6582

ΣFAs = sum of all FAs; FAME = fatty acid methyl ester; SA = stearic acid; LA = linoleic acid; ALA = linolenic acid; ARA=arachidonic acid; EPA = eicosapentaenoic acid; DPA = docosapentaenoic acid; DHA = docosahexaenoic acid. SFA = sum of the individual saturated fatty acids; UFA = sum of the individual unsaturated fatty acids; MUFA = sum of the individual monounsaturated fatty acids; PUFA = sum of the individual polyunsaturated fatty acids; OCFA = odd-chain fatty acids; BCFA = branched-chain fatty acids, sum of iso- and anteiso-FA; OBCFA = odd- and branched-chain fatty acids, sum of odd-, iso-, and anteiso-FA; SCFA, short-chain fatty acids (sum of individual fatty acids from C4:0 to C10:0); MCFA = medium-chain fatty acids, sum of the individual fatty acids from C11:0 to C17:0; LCFA = long-chain fatty acids, sum of the individual fatty acids from C18:0 to DHA; PUFA n-3 and PUFA n-6 = sum of individual n-3 and n-6 fatty acids, respectively; CLA = sum of individual conjugated linoleic acids; TI = thrombogenic index; AI = atherogenic index; h:H = hypocholesterolemic to hypercholesterolemic ratio.
